# Supplementary figures and images for: Description and rediagnosis of the crested hadrosaurid (Ornithopoda) dinosaur Parasaurolophus cyrtocristatus on the basis of new cranial remains
Source: PeerJ. 2021 Jan 25;9:e10669. doi: 10.7717/peerj.10669 (PMC7842145; doi:10.7717/peerj.10669)

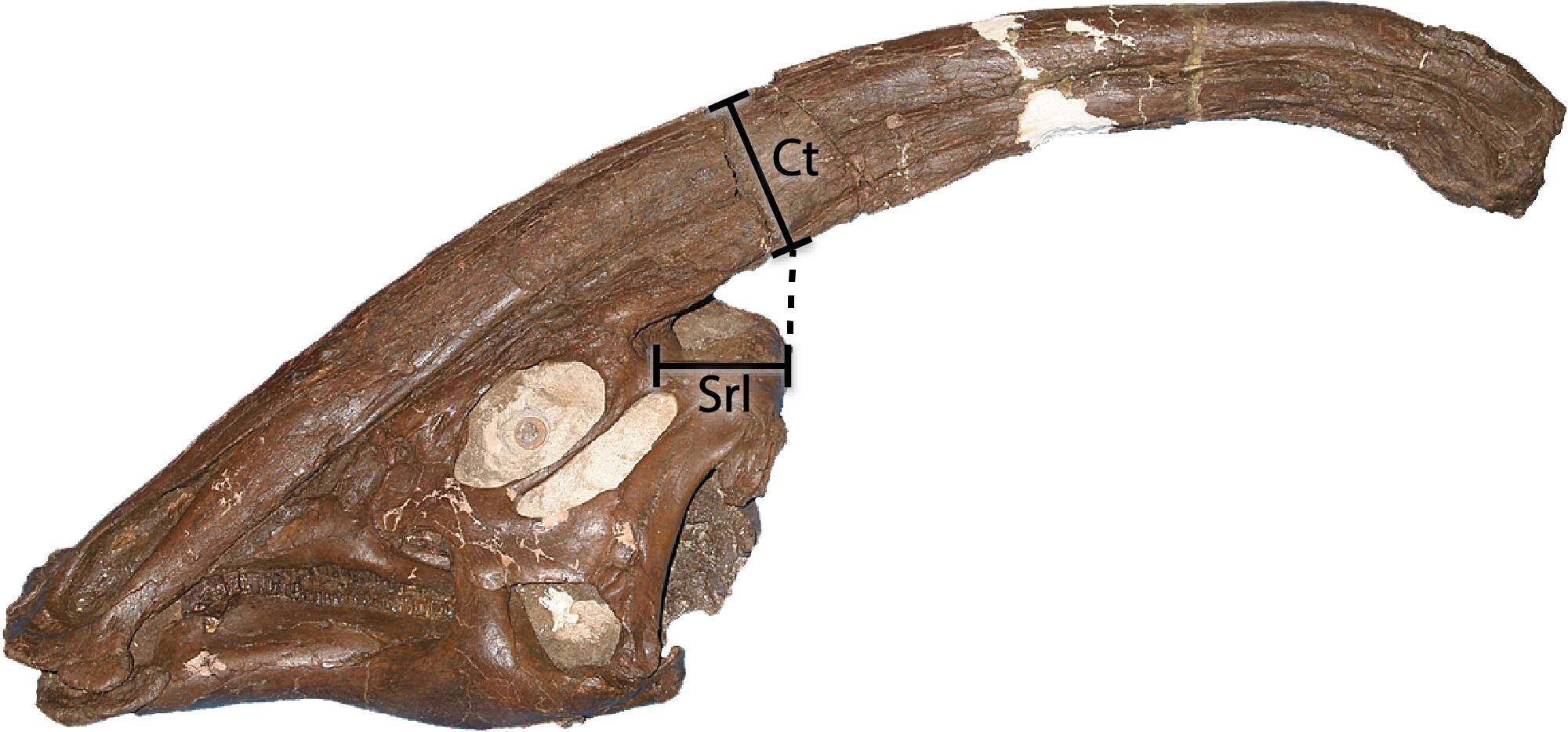

Supplement: Supplemental Information 3 [file peerj-09-10669-s003.png]
