# Supplementary material for: Description and rediagnosis of the crested hadrosaurid (Ornithopoda) dinosaur Parasaurolophus cyrtocristatus on the basis of new cranial remains
Source: PeerJ. 2021 Jan 25;9:e10669. doi: 10.7717/peerj.10669 (PMC7842145; doi:10.7717/peerj.10669)
Supplement: Supplemental Information 4 [file peerj-09-10669-s004.docx]

List of measurements supporting crest thickness character in phylogenetic analysis. All measurements made in ImageJ and reported in millimeters.

|  | Crest Thickness (Ct) | Skull Roof Length (Srl) | Ct/Srl Ratio |
| --- | --- | --- | --- |
| *Parasaurolophus walkeri* (ROM 768) | 127 | 127 | 1 |
| *P. cyrtocristatus* (FMNH P-27393) | 141 | 143 | 0.99 |
| *P. tubicen* (NMMNH P-25100) | 191 | 121 | 1.57 |
